# The Green Paper. An Hypothesis to Explain the Delay in Its Appearance

**Published:** 1986-08

**Authors:** Alick Dowling

**Affiliations:** General Practitioner, Bristol


### The Green Paper. An Hypothesis to explain the delay in its appearance

**Alick Dowling M.B., Ch.B.**
General Practitioner, Bristol

In the DHSS, the Permanent Secretary, Sir Horace Appleby (brother of the better known Sir Humphrey) and Nigel (The Minister's Private Secretary) await the arrival of the new Minister of Health at his first appearance in the Department offices.
*Sir Horace:* All ready to meet your new Minister, Nigel?
*Nigel:* Yes, Sir Horace; but they do seem to change rather frequently and this one seems to be a very different character from the last one.
*Sir Horace:* There are two main types who are appointed to this Ministry, which has become a traditional transitory posting, type A, the young, thrusting, ambitious man anxious to show his potential and looking for early promotion; and type B the politician on his way down, either towards retirement or after relative failure in some other post, not sufficient to merit dismissal. Both can be 'guided' to aid our long term strategy.
*Nigel:* You mean that we can persuade this one also to follow *our* policy?
*Sir Horace:* Exactly. While the recent incumbent, though seemingly difficult and obstinate, not to say self-opinionated was admirable in initiating controversial policies that had the great advantage of really stirring up the Medical Profession, the new man will give the optimists amongst the doctors the hope that things will change for the better, while we know that the policies that are concerned with increasing control of the Medical Profession are in so far an advanced state of preparation that it would be impractical to alter them now.
*Nigel:* But surely the appointment of this particular Minister was presented to the Medical Profession as a sort of olive branch after the recent controversies about deputising and the limited list.
*Sir Horace:* Of course. But that does not mean that we cannot carry on as before.
*Nigel:* You mean, pretend that nothing has changed.
*Sir Horace:* No pretence about it; nothing *has* changed as far as we are concerned. At least nothing of significance.
*Nigel:* But surely Sir Horace, the Minister will want to impress the doctors with his concern for them and their problems.
*Sir Horace:* No harm in allowing him to indulge his compassion, provided that we do not let him try to reverse matters that are already decided.
*Nigel:* But I thought all this was still up for discussion; after all it is only a Green Paper that we are publishing.
*Sir Horace:* Surely Nigel, you're not so naive as to believe that what is in the Green Paper can really be substantially changed at this stage in an Administration.
*Nigel:* You mean that if it's not implemented fairly soon, it won't get Parliamentary assent before the next election.
*Sir Horace:* You're learning Nigel. We use the electoral timetable to suit our purposes; sometimes delaying, sometimes pressing forward, depending on whether what a Minister currently favours suits our long-term strategy or not.
*Nigel:* But surely Sir Horace, if we have a Minister just appointed, and with the brief to mollify the doctors for example, how can we really divert him from what he will see as his duty.
*Sir Horace:* We encourage him; after all it is part of *our* policy now to 'mollify the doctors' as you put it; after so much conflict a conciliatory approach will unsettle the realists who know our aims, and at the same time disarm the less informed.
*Nigel:* You mean the gullible.
*Sir Horace:* Not a description to be used outside these walls, but yes I think that is a fair way of delineating those members of the profession who want to like us.
ENTER THE MINISTER:
*Sir Horace:* Good-morning Minister; I believe you've already met Nigel, your new Principal Private Secretary.
*Minister:* Yes, indeed. Good-morning Nigel.
*Nigel:* Good-morning Minister. Have you had a chance yet to look at the file I left you?
*Minister:* Indeed yes; I found the Green Paper very challenging.
*Sir Horace:* A revealing adjective, Minister; I hope that you don't feel it needs any substantial amendment.
*Minister:* Well, yes, there are a number of things that need changing before it is published. In particular, I'm not very happy with all those ideas that the Medical Profession are bound to see as punitive.
*Sir Horace:* Certainly Minister; if you will let me have a copy of the proposals you would like altered, I will arrange for them to be redrafted.
*Minister:* That isn't quite what I had in mind. I intended to redraft them myself; after all I've had quite a lot of experience in this field. How long have we got before publication date?
*Sir Horace:* Well they were most recently promised for before Christmas.
*Minister:* I don't think that would be a good idea. Remember the terrible reception that the deputising restrictions received when they were promulgated just before Christmas two years ago. The doctors regarded that as a provocative piece of timing. And the introduction of the restricted list on April Fool's day wasn't exactly tactful. We don't want to repeat those mistakes do we?
*Sir Horace:* If you say so Minister. But if we leave it too long we shall run into trouble with the Parliamentary timetable.
*Minister:* We should have plenty of time, particularly if we can agree on a set of proposals that will command the support of the Profession.
*Sir Horace:* Not much chance of that I'm afraid Minister with them in their present mood.
*Minister:* My aim, indeed my appointment was designed to change all that.
*Sir Horace:* I trust Minister, that you are aware that the 'controversies' as you call them of the last two years, have substantially improved our position with regard to control of the Profession.
*Minister:* What exactly do you mean?

*Sir Horace:* Well, for a start, we have created a notable, though so far largely unnoticed, precedent in altering their terms of service without the tedium of having to negotiate this with their elected representatives.
*Minister:* Explain.
*Sir Horace:* We've imposed limitations on use of deputising services through the new so-called independent FPCs which are largely arbitrary and lack validity.
*Minister:* I thought that a breach in terms of service was something for which doctors could be severely penalized?
*Sir Horace:* Yes, but that doesn't apply when it is we who are doing the breaching. Anyway it's been tacitly accepted by the Profession. And there are no equivalent penalties in the regulations that would apply to us.
*Minister:* I didn't suppose there would be. What other devious things have you been doing?
*Sir Horace:* Nothing devious Minister. We have been keeping an eye on the limited list of prescribable drugs. A few alterations here and there, to perpetuate uncertainty; this has a satisfyingly unsettling effect—keeps everyone on their toes.
*Minister:* And saves money I hope, and also improves things for patients.
*Sir Horace:* Of course—but was there anything interesting discussed at the meeting of Ministers today?
*Minister:* Nothing relevant to the NHS but there was a lot of argument about the Westland helicopter firm's future; as you've probably heard, a substantial difference of opinion has emerged. On one side are those who favour a European takeover, largely French, while on the other there is an American offer supported by Fiat. I don't know which to support. What do you think Horace?
*Sir Horace:* Not much of a choice is it? The Frogs or the Mafia. What does the PM say?
*Minister:* Oh!, the official line is that it is up to the shareholders to decide. But they have as much chance of understanding what's at stake as a jury in a fraud trial. What beats me is why anyone should want to take over an inefficient overmanned West Country garage; but there we are; I don't know which side to support. And yet I need to be on the winning side; there's a danger of appearing a ditherer if I delay deciding much longer.
*Sir Horace:* Could I suggest Minister that you assume the mantle of your new office—that of 'The Healer', the resolver of disputes, the non-partisan umpire above the petty squabblers.
*Minister:* That sounds attractive.
*Sir Horace:* But of course you must find out which side the Treasury are on and, as umpire, manipulate a decision in accord with their wishes.
*Minister:* But why does it matter what they think; they've no expertise on this?
*Sir Horace:* That's not the point; you now represent a high spending department; it is your task to ensure that funds for our programmes have priority over other claims; it is politic to support the Treasury when it doesn't conflict with our own interests.
*Minister:* You mean they'll then finance the costs involved in our new proposals?
*Sir Horace:* Hopefully yes. But we mustn't anticipate added expenditure; economy is their main raison d'être. Apart that is of course from our usual increase of control over the Medical Profession.
*Minister:* I've never heard of any proposals that have yet produced their planned savings.
*Sir Horace:* You mustn't be cynical Minister. there is a good chance that by insisting on compulsory retirement age for GPs, stopping the 24 hour retirement scheme, and further tightening the screw on deputising restrictions and on the limited list will actually give us savings.
*Minister:* But these are precisely the things I want to alter in the Green Paper before it is published. I mustn't start off on the wrong foot with the doctors as my predecesor did. I'll let you have my amended proposals as soon as I can. I've got to get back to the House now. EXITS.
*Nigel:* He seems very determined to have his own way, Sir Horace, for a type B appointment. What do we do now?
*Sir Horace:* Bide our time. The changes already instituted will prove impossible for any Minister to stop, especially a type B.

